# Fistula of the Matrix

**Published:** 1896-08

**Authors:** T. A. J. Beel


Fistula of the Matrix. By T. A. J. Beel. On the 17th of September, 1895, my aid was asked in the case of a milch cow about four years old which had a swelling in the region of the left groin. Round about the swelling an œdema was noticeable which extended as far as the median line of the belly and further toward the front. Inasmuch as at least in one place abscesses with a break behind them (opening) were present, I concluded not to open them for the present, but pursue an expectant course. The swelling itself was anointed with laurel salve. On the following day it had become twice as large. The œdema had spread over the whole left half of the belly to behind the elbow and was hot and very painful. A puncture, repeated twice, of apparently fluctuating spots yielded no result. The left half of the udder was very much swollen. But all remained to the left of the median line. The treatment was continued until the 23d of September. Then the swelling increased a little, while movement on the part of the animal was painful and stiff, and evacuation had almost ceased. After Priesnitz's poultices had been applied for two days without result, suddenly with increase of the swelling, that well-known crackling (rumble) of gases under the hide began, originating in the region of the groin where the swelling had its origin and extending over the shoulder-blade, neck, etc. Besides this the dashing (trickle) of fluid could be noticed. The owner decided to kill, and the post-mortem examination resulted as follows: The skin over the left half of the belly could hardly be loosened from the swelling beneath. Where this close connection was severed by the knife, a number of little yellow knobs could be seen about the size of millet-corn, apparently little tubercles. When the skin had been entirely removed the swelling was blue and variegated. A section lengthwise, about four fingers deep, showed a spongy tissue containing air, resembling a transverse section of a lung which had been partly drained of blood. Here also, from the cavities, which were of varying dimensions, fluid and foam bubbled up here and there. Between these cavities larger and smaller tubercles were found. After this thick layer had been passed a large cavity containing fluid was found, out of which about 15 litres of reddish-yellow, odorless, clear liquid flowed. It had evidently been collected between two layers of muscles, and the former puncture had failed of its purpose because the trocar did not penetrate deep enough. On opening this cavity a large quantity of odorless gas escaped. In order to gain a better view of the conditions it was decided to open the abdominal cavity first. The abdominal organs were normal, very anæmic; the spleen somewhat enlarged. The matrix attracted attention at once. It looked dark-red, was soft to the touch, and the left horn, which was larger than normal, had become adherent to the surface of the abdomen which corresponded to the swelling. On closer examination the left ovary appeared to be enlarged by a tuberculous process which evidently caused the adhesion between the horn of the uterus and the abdominal wall. The uterus on being opened lengthwise showed thickened walls, while the surface had a gloss-like appearance. The inner cavity contained liquid similar to that in the cavities mentioned above. Where the uterus and abdominal wall joined there was a large opening through which two fingers could pass, the edges were entire, and it communicated with the accumulations of fluid between the different layers of muscles. The fluid was thus produced in the matrix and passed through the fistula toward the outside. That gas had found its way later into the swelling is explained also by the passage of gas through the mouth of the matrix, which appeared, even at the post-mortem, not entirely closed. The whole course of the disease can be imagined as follows: The left ovary had first been infected with tuberculosis, through which the attachment to the abdominal wall was brought about and the spread of tuberculosis to the abdominal muscles. At the place where the two walls had grown fast together a tuberculous abscess formed which discharged toward the matrix and left behind the opening about the size of two fingers. The diseased wall of the matrix, which secretes liquid under such conditions, furnished also in this case the large quantity of fluid stuff which found an outlet for itself through this opening and so flowed in between the muscular layers. That these were tuberculously affected at several places and had grown together explains also why there were several cavities in which fluid stuff was present. The apparent improvement in the condition of the animal at times is perhaps to be explained by the flowing back of the fluid from the cavities to the matrix whenever circumstances favored it, as, for example, in lying down. when it could easily take place in consequence of the increased pressure on the upper surface of the fluid. That both the fluid and gases were odorless was explained through the entrance of air from without shortly before. The mouth of the matrix was evidently closed for a long time, but finally opened in consequence of the general weakness of the body.—*Tijdschrift voor Veeartsenijkunde*, January, 1896.

